# IGF-1 Increases with Hyperbaric Oxygen Therapy and Promotes Wound Healing in Diabetic Foot Ulcers

**DOI:** 10.1155/2013/567834

**Published:** 2013-02-26

**Authors:** Figen Aydin, Ahmet Kaya, Levent Karapinar, Mert Kumbaraci, Ahmet Imerci, Hasan Karapinar, Cengiz Karakuzu, Mustafa Incesu

**Affiliations:** ^1^Neoks Hyperbaric Oxygen Therapy Center, Bornova, 35560 Izmir, Turkey; ^2^Underwater and Hyperbaric Medicine, Yeni Girne Bulvari No. 211, Karsiyaka, 35550 Izmir, Turkey; ^3^Department of Orthopaedics and Traumatology, Tepecik Education and Research Hospital, Yenisehir, 35100 Izmir, Turkey

## Abstract

*Objectives*. To investigate insulin-like growth factor I (IGF-1) levels in response to hyperbaric oxygen therapy (HBOT) for diabetic foot ulcers and to determine whether IGF-1 is a predictive indicator of wound healing in patients with diabetic foot ulcers. *Design and Methods*. We treated 48 consecutive patients with diabetic foot ulcers with HBOT. Alterations of IGF-1 levels in patients whose wound healed with HBOT were compared with those in patients who did not benefit from HBOT. *Results*. There was no significant difference in initial IGF-1 levels between the two groups (*P* = 0.399). The mean IGF-1 level increased with HBOT (*P* < 0.05). In the healed group, the mean IGF-1 increase and the final values were significantly higher (*P* < 0.05). In the nonhealed group, the mean IGF-1 increase was minus and the final values were not significantly different (*P* < 0.05). The increase in IGF-1 level with HBOT was significantly higher in the healed group (*P* < 0.001). *Conclusions*. IGF-1 increased significantly in the healed group. We believe that HBOT is effective in the treatment of diabetic foot ulcers, with an elevation of IGF-1. This alteration seems to be a predictive factor for wound healing in diabetic foot ulcers treated with HBOT.

## 1. Introduction

Wound healing has been shown to be delayed in patients with diabetes and particularly in patients with diabetic foot ulcers [1]. Peripheral neuropathy, peripheral vascular disease, and poor glycemic control in conjunction with minor foot trauma increase the likelihood that patients with diabetes will develop foot ulcers [2]. Between 5 and 10% of diabetic patients have or have had such foot ulcerations, with approximately 1% requiring lower extremity amputation. The risk of amputation for those patients is greater than for the nondiabetic population [1, 3]. 

Growth factors influence the healing process, and among these, insulin-like growth factor (IGF) has been shown to stimulate keratinocyte proliferation *in vitro* [1, 4]. Existing evidence indicates that IGF-1, a proinsulin-like growth factor that modulates tissue growth and repair, and IGF-binding proteins (IGFBPs) play important roles in glucose homeostasis in diabetic patients [1, 5–7]. A lack of IGF-1 expression within the basal layer and fibroblasts may contribute to retarded wound healing in diabetes mellitus patients [1]. 

Insulin and insulin-like growth factor (IGF-1) play important roles in vascular biology. Both hormones regulate vascular tone, in part by decreasing vasoconstrictor responses to agonists, such as angiotensin II, norepinephrine, and vasopressin [5].

 There are two isoforms of IGF in mammals, and they have been implicated in the pathogenesis of diabetic complications. A few studies have been performed on the distribution of these growth factors during wound healing in diabetic animals, but no studies have been carried out in human subjects. The levels of IGF-1 in wound fluid and plasma are diminished in diabetic patients, but the latter finding is not consistent in the literature. It is not clear whether plasma levels of IGF-2 change in diabetes, but like IGF-1, the level of IGF-2 in wound fluid is decreased [1]. 

 HBOT, which delivers 100% oxygen at pressures above one atmosphere, is thought to assist wound healing, as it delivers a significantly increased amount of oxygen to the skin and surrounding tissues [8]. These growth-promoting effects suggested that HBOT may be used as an adjuvant to promote collagen synthesis and improve wound healing for diabetic venous, arterial, and pressure ulcers [1]. It has been suggested that the use of adjunctive HBOT will improve the healing of diabetic lower leg ulcers and decrease the risk of major lower extremity amputation [2, 3] HBOT has been used as a treatment for various chronic and acute wounds [8], and it has significantly reduced the risk of major amputation and improved the chance of healing [3, 8].

To our knowledge, in the literature, there is little information on the relationship between IGF-1 and wound healing in diabetic foot ulcers treated with HBOT.

In the present study, we aimed to determine whether IGF-1 levels were changed in response to HBOT in diabetic foot ulcers. We also investigated whether IGF-1 was a predictive indicator of wound healing in diabetic foot ulcers treated with HBOT.

## 2. Materials and Methods

Between January 2008 and January 2009, 48 consecutive patients with diabetic foot ulcers were treated with HBOT as an adjunct to standard treatment modalities. On admission, the wounds were classified according to Wagner. The diabetes mellitus type, duration of diabetes, type of diabetes treatment, age of diabetic wound, previous diabetic ulcer history, diabetic foot deformity, and smoking habits were recorded. Glycosylated hemoglobin and IGF-1 levels were measured. Consultations were performed to determine neuropathy, nephropathy, and retinopathy. Specimens were collected from foot lesions for culture and antimicrobial susceptibility tests.

 Initially, aggressive debridement was performed, and the wound was dressed. Debridements whenever needed were repeated.

Dressings were changed at required intervals. After the collection of swabs from the wound, patients were given empirical antibiotic treatment (Cephalosporin and quinolones). This treatment was modified if necessary according to the sensitivity tests. 

Blood glucose levels were optimized with insulin. The feet were protected from uncontrolled mechanical stresses. Subsequently, the patients underwent HBOT. The inclusion criteria were Wagner grades II, III, IV, and V diabetic foot ulcers wounds. Exclusion criteria were pneumothorax, cardiopulmonary failure, malignancy, and claustrophobia. 

HBOT was performed in a multiplace chamber (Hiperbot Model 101, 2005, Turkey), which makes it possible to treat ten patients simultaneously. First, the chamber was pressurized with compressed air for 15 min. When the pressure in the chamber reached a level equivalent to 42 feet (14 m) depth, the patients breathed 100% oxygen using a mask. Thus, they were exposed to 2.4 absolute atmosphere (ATA) pressures when breathing 100% oxygen. Each therapy session consisted of three oxygenation periods. These periods lasted 25 min each and were separated by 5 min air breaks. Subsequently, the chamber was decompressed for 15 min, and the therapy was complete. Therefore, one treatment session took approximately 120 min. Each patient received 30 total sessions of HBOT. A member of the medical staff accompanied the patients in the chamber during the entire session. The HBOT sessions were repeated once daily for six days a week. 

The results were evaluated as healed and nonhealed (not improved and amputated).

At the end of HBOT, glycosylated hemoglobin and IGF-1 levels were measured again. The initial and final values were compared for both tests, and alterations were noted. IGF-1 alterations in the healed group and nonhealed groups were also statistically compared to determine whether an increase in IGF-1 is a predictive factor of wound healing in diabetic foot ulcers. The other clinical and laboratory tests were also compared from the beginning to the end of treatment and between the healed and nonhealed groups. Healing was assessed at the end of HBOT and the follow-up examinations were repeated at the end of third, sixth, and 12th month.

Descriptive statistics, the chi-square test, Fischer's exact test, the Mann-Whitney  *U*  test, linear regression analysis, two-sided analyses, and multiple comparison tests for univariate analysis were performed where appropriate. SPSS version 13 was used for statistical analysis.

## 3. Results

Of all patients, 37 (77.1%) were male, and 11 (22.9%) were female. The mean age was 59.19 (±11.10) years. The clinical characteristics of the patients are shown in Table 1. 

The wound classifications according to the Wagner scale were as follows: no Wagner grade 1, 2 (4.2%) grade 2, 30 (62.5%) grade 3, 14 (29.2%) grade 4, and 2 (4.2%) grade 5.

The wound classification according to the healed and nonhealed groups was as follows: healed group: no grade 1, 2 (5%) Wagner grade 2, 25 (62.5%) grade 3, 12 (30%) grade 4, and 1 (2.5%) grade 5; nonhealed group: no grade 1 or 2, 5 (62.5%) grade 3, 2 (25%) grade 4, and 1 (12.5%) grade 5. There was no statistically significant difference between these two groups according to the Wagner classification (*P* = 0.58).

The mean Wagner grade at admission was 3.33 ± 0.63 overall. In the healed group, the mean initial Wagner grade was 3.30 ± 0.60, and in the nonhealed group, the mean Wagner grade at admission was 3.50 ± 0.75. There was no statistically significant difference between the groups (*P* = 0.560).

There were no differences between healed and nonhealed groups in terms of culture test. *Staphylococcus aureus* (*n* = 14) and *Pseudomonas aeruginosa* (*n* = 14) were the main pathogenic organisms before the HBOT. The remaining pathogenic organisms were *Enterococcus* (*n* = 6), *E. coli* (*n* = 4) and *Klebsiella* (4). In 6 cases the culture tests were negative. Although the culture tests remained positive in wounds which are infected by *Pseudomonas aeruginosa*, the healing was obtained in 2 cases. On the other hand, the healing could not be obtained in some cases although the culture tests turned to negative from positive. We could not find any relations between the IGF-1 levels and wound infection.

Peripheral vascular diseases (PVH) were diagnosed in 18 cases with colored doppler ultrasonography and/or angiography. Vascular surgeon did not need surgical interventions for these patients. 3 of 8 nonhealed patients and 15 of 40 healed patients had peripheral vasculopathy.

There were no significant differences in terms of patient age, gender, duration of diabetes mellitus, or initial HbA1C values between the healed and nonhealed groups (Table 2).

At the end of the treatment, 40 patients (83.33%) (30 males (81.1%) and 10 females (90.9%)) were healed, and the remaining 8 patients (16.67%) (7 males (18.9%) and 1 female (9.1%)) showed no improvement and/or underwent amputation.

In our study, we did not observe any permanent complications or patient compliance problems. The patients experienced some mild side effects. The most common complaint with HBOT was middle ear barotraumas. None of these patients interrupted or withdrew from HBOT.

There was no significant difference in IGF-1 changes from initial to final values in terms of patient age, duration of diabetes mellitus, Wagner grade, initial HbA1C value, final HbA1C value, or HbA1C alterations (*P*  values were 0.93, 0.46, 0.76, 0.88, 0.70, and 0.40, resp.). 

The initial and final values and alterations in IGF-1 are shown in Table 3.

## 4. Discussion

Diabetic foot ulcers are a common and serious complication of diabetes [9, 10]. Treatment often requires long-term hospital admissions and frequent outpatient visits. Furthermore, loss of mobility imposes a great burden on the patient and the health care system [8]. At excellent centers, 19–35% of ulcers are reported as nonhealed [5–7]. Thus, despite improvements in healing diabetic foot ulcers, there is still a need for new treatment strategies and methods. Systemic hyperbaric oxygen therapy (HBOT) has been proposed as a medical treatment for diabetic foot ulcers [11]. HBOT has been demonstrated to have an antimicrobial effect and increase the oxygenation of hypoxic wound tissues [12–14]. This enhances neutrophil killing ability, stimulates angiogenesis, and enhances fibroblast activity and collagen synthesis [14–16]. Thus, HBOT could improve the healing of ischemic foot ulcers in patients with diabetes, [17, 18].

Elevated oxygen pressure in the plasma causes the upregulation of growth factors, downregulation of inflammatory cytokines, increased fibroblast activation, angiogenesis, antibacterial effects, and enhanced antibiotic action. Consequently, it has been used in the treatment of these wounds since the 1980s [3].

Evidence from animal and cell line studies has shown that HBOT, the administration of pure oxygen at pressures greater than 1 atmosphere absolute (ATA), results in increased growth factor production, such as platelet-related growth factor, transforming growth factor-*β*  1, vascular endothelial growth factor, and reactive oxygen species-related hypoxia-inducible factor-1, in wound healing [10]. 

Based on the localization of IGF in diabetic and nondiabetic endothelial cells, it may be speculated that IGF plays a role in angiogenesis. As previously stated, IGF-1 is a chemo-attractant for endothelial cells, but this hypothesis needs further study [1].

In healthy subjects, serum IGF-I levels peak in early adulthood, after which they gradually decrease with increasing age. Several observations suggest that there is a premature and progressive age-related decline in serum IGF-I bioactivity in type 2 diabetics, which eventually results in a (relative) IGF-I deficiency. In type 2 diabetics, close relationships have been demonstrated between glycemic control and serum IGF-I levels, with worse control being associated with lower IGF-I levels. IGF-I is most likely the most potent anticatabolic and anabolic hormone in the body. In patients with type 2 diabetes, serum IGF-I levels are dependent on the degree of metabolic control, with almost normal IGF-I levels in well-controlled diabetics, whereas the levels tend to decrease in poorly controlled diabetics. It has also been suggested that decreased serum IGF-I concentrations predict the worsening of insulin-mediated glucose uptake in older people. In type 2 diabetes, this progressive age-dependent decline of total IGF-I levels is even greater than observed in healthy controls. All of these findings suggest that in type 2 diabetes, during aging, there is a premature and progressive decline in serum IGF-I bioactivity, resulting in the development of a progressive (relative) IGF-I deficiency, especially in those with poor metabolic control, which can cause vascular damage and vascular dysfunction. Vascular dysfunction is associated with all major risk factors for atherosclerosis. Circulating IGF-I, like insulin, stimulates nitric oxide (NO) in certain vascular beds. Reduced NO generation or accelerating NO inactivation may cause vascular damage and vascular dysfunction [16]. 

In conclusion, the lack of IGF-1 and the elevated levels of IGF-2 within the basal layer of the epidermis of diabetic patients may be important in delayed wound healing in diabetes mellitus, particularly for chronic foot ulcers [1].

In the present study, we aimed to investigate alterations of IGF-1 after HBOT for diabetic foot ulcers to determine whether it could be a predictive factor of wound healing with HBOT. Despite numerous studies, limited information exists on the role and efficacy of HBOT. Furthermore, to our knowledge, there is little information about the efficacy of HBOT and IGF-1 in human subjects. 

In our study, we achieved good results with HBOT for most of the patients (40/48, 83.33%) with diabetic foot ulcers. 

We also observed a significant IGF-1 increase after HBOT, which promotes wound healing. When we stratified the results as healed and nonhealed, there was no significant difference between groups in terms of the initial IGF-1 values. We also did not observe a significant difference in terms of patient age, gender, duration of diabetes mellitus, initial HbA1C values, or Wagner wound classification.

When we statistically analyzed the IGF-1 alterations according to age, duration of diabetes mellitus, Wagner classification, initial and final HbA1C values, and HbA1C alterations, we could not find a significant difference.

IGF-1 values increased to an average of 201.12 ± 66.93 from 152.36 ± 92.69 at the end of HBOT. The difference was statistically significant (*P* < 0.05). This result could show the efficacy of HBOT in diabetic foot ulcers. In the healed group, the average IGF-1 values increased to 216.91 ± 92.12 from 155.73 ± 70.00 (*P* < 0.05), and in the nonhealed group, the average IGF-1 values decreased to 122.16 ± 43.07 from 135.50 ± 48.82 (*P* > 0.05). The difference between these two groups was highly significant (*P* < 0.001), although there was no significant difference between initial values. This result led us to suggest that IGF-1 values could be a predictive factor of wound healing in diabetic foot ulcers treated with HBOT.

This study has some weak points. The most important limitation is the absence of a control group. Thus, randomization could not be performed. Moreover, strict conclusions on direct HBOT efficacy could not be reached. However, it is difficult to apply sham therapy; in our clinical conditions, that would pose a problem with receiving payment from the social security company. Because our institute has no HBOT unit, we used a private HBOT center to treat our patients. The HBOT center and clinicians were blinded to the other part of the treatment and evaluation. The number of cases is relatively high, and the follow-up period is sufficient. The study group consisted of consecutive patients admitted to our clinic, and no selection of cases was applied. Approximately two-thirds of the patients were Wagner grades 3 and 4, and the study group involved all Wagner grades (except grades 0 and 1).

## 5. Conclusion

In conclusion, we observed that with HBOT we found an increase in IGF-1 levels, which promotes wound healing. Although there were no significant differences in initial IGF-1 values between healed and nonhealed groups, the statistically significant increase in the healed group led us suggest IGF-1 as a predictive factor for wound healing in diabetic foot ulcers treated with HBOT.

## Figures and Tables

**Table 1:** Clinical characteristic of the patients.

	Minimum	Maximum	Mean ± SD
Age (years)	29	83	59.19 ± 11.10
Healed group	29	83	60.20 ± 10.72
Non-healed group	38	72	54.13 ± 12.35
Duration of the wound (weeks)	3	248	23.88 ± 51.31
HbA1C (initial) (mg/100 mL)	5.1	14.7	8.13 ± 2.13
HbA1C (at the end of treatment) (mg/100 mL)	5.3	9.3	7.041 ± 1.15

		*N*	%

Sex			
Male		37	77.1
Healed		30	81.1
Non-healed		7	18.9
Female		11	22.9
Healed		10	90.9
Non-healed		1	9.1
Type of DM			
Type 1 DM		1	2.08
Type 2 DM		47	97.92
Wagner classification of wounds			
Grade 2		2	4.2
Grade 3		30	62.5
Grade 4		14	29.2
Grade 5		2	4.2

DM: diabetes mellitus, HbA1C: glycosylated hemoglobin, OAD: oral antidiabetic drug, HBOT: hyperbaric oxygen therapy, SD: standard deviation.

**Table 2:** Comparison of the clinical parameters between the non-healed/amputated and healed group.

Clinical parameter	*P* value
Wagner grade	0.580
Age	0.213
HbA1C initial	0.870
Sex	0.661
DM duration	0.922
IGF-1 initial	0.399

DM: diabetes mellitus, HbA1C: glycosylated hemoglobin, IGF-1: insulin-like growth factor 1.

**Table 3:** Summary of IGF-1 values.

IGF-1	Minimum	Maximum	Mean ± SD
Initial values			
Overall	48	377	152.36 ± 66.93
Healed	48	377	155.73 ± 70.00
Non-healed	85	233	135.50 ± 48.82
IGF-1 final values			
Overall	57	480	201.12 ± 92.69
Healed	57	480	216.91 ± 92.12
Non-healed	80	180	122.16 ± 43.07
IGF-1 response to HBOT			
Overall	−55	+276	48.75 ± 62.10
Healed	−25	+276	61.17 ± 59.84
Non-healed	−55	+25	−13.33 ± 25.67
IGF-1 percentage (final/initial)			
Overall	0.68	2.90	1.37 ± 0.49
Healed	0.86	2.90	1.46 ± 0.49
Non-healed	0.68	1.29	0.91 ± 0.19

Statistics			
Parameter		Healed*/*nonhealed	

IGF-1 initial		*P* = 0.399	
IGF-1 response to HBOT		*P* < 0.001	
IGF-1 percentage (final value/initial value)		*P* < 0.001	
IGF-1 response to HBOT and IGF-1 percentage (final/initial)			
Age		*P* = 0.93	
Duration of DM		*P* = 0.46	
Wagner grade		*P* = 0.76	
HbA1C initial		*P* = 0.88	
HbA1C final		*P* = 0.70	
HbA1C response to HBOT and percentage		*P* = 0.40 and 0.37, respectively	
